# Differential Gene Expression from Genome-Wide Microarray Analyses Distinguishes Lohmann Selected Leghorn and Lohmann Brown Layers

**DOI:** 10.1371/journal.pone.0046787

**Published:** 2012-10-08

**Authors:** Christin Habig, Robert Geffers, Ottmar Distl

**Affiliations:** 1 Institute for Animal Breeding and Genetics, University of Veterinary Medicine Hannover (Foundation), Hannover, Germany; 2 Department of Cell Biology, Helmholtz Centre for Infection Research, Braunschweig, Germany; Rutgers University, United States of America

## Abstract

The Lohmann Selected Leghorn (LSL) and Lohmann Brown (LB) layer lines have been selected for high egg production since more than 50 years and belong to the worldwide leading commercial layer lines. The objectives of the present study were to characterize the molecular processes that are different among these two layer lines using whole genome RNA expression profiles. The hens were kept in the newly developed small group housing system Eurovent German with two different group sizes. Differential expression was observed for 6,276 microarray probes (FDR adjusted P-value <0.05) among the two layer lines LSL and LB. A 2-fold or greater change in gene expression was identified on 151 probe sets. In LSL, 72 of the 151 probe sets were up- and 79 of them were down-regulated. Gene ontology (GO) enrichment analysis accounting for biological processes evinced 18 GO-terms for the 72 probe sets with higher expression in LSL, especially those taking part in immune system processes and membrane organization. A total of 32 enriched GO-terms were determined among the 79 down-regulated probe sets of LSL. Particularly, these terms included phosphorus metabolic processes and signaling pathways. In conclusion, the phenotypic differences among the two layer lines LSL and LB are clearly reflected in their gene expression profiles of the cerebrum. These novel findings provide clues for genes involved in economically important line characteristics of commercial laying hens.

## Introduction

Microarray expression profiling became a universal tool, with a range of applications that benefit from the accurate determination of differential gene expression [1], [2]. It allows simultaneous measurement of the expression levels of thousands of genes in a single hybridization experiment. Microarray technologies have been proven to be valuable for understanding biological pathways important in various physiological processes [3]. Due to increasing importance of genome research in livestock species, microarray resources are now available for many agriculturally important species [3] including the chicken. The chicken is an important model organism for evolutionary and developmental biology, immunology, genetics and agricultural science [4]. Buitenhuis et al. [5] explored differentially expressed genes for aggressive pecking behaviour in laying hens from a high feather pecking selection line produced from a foundation stock of a White Leghorn layer strain. They compared genome-wide profiles of chicken brain samples from aggressive and receiver hens using a 20 K chicken microarray that contained 20,678 oligonucleotides and corresponded to 20,640 chicken transcripts. They detected a number of gene ontology (GO) identifiers which are potentially involved in aggressive behavioural processes, including genes encoding for synaptosomes and proteins involved in the regulation of the excitatory postsynaptic membrane potential, the regulation of the membrane potential, and glutamate receptor binding. Similarly, Brunberg et al. [6] used Affymetrix GeneChip® Chicken Genome Arrays to compare gene expression on Lohmann Selected Leghorn hens performing feather pecking and those receiving feather pecking. Among the differently expressed genes they found genes involved in disorders, such as intestinal inflammation and insulin resistance.

One of the worldwide leading breeding companies for laying hens is Lohmann Tierzucht (Cuxhaven, Germany). The Lohmann Selected Leghorn (LSL) layer line and the Lohmann Brown (LB) hens have been firmly established in most markets of the world because of their efficient production of high quality white and brown eggs, respectively [7]. Despite their approximately identical egg production performance these layer lines differ markedly in other phenotypic traits. Behavioural studies observed differences in pecking activity between LSL and LB at 38 weeks of age, whereas total number of peeks and bouts were higher in LB [8],[9]. A number of studies showed that LSL layers have lower humerus breaking strengths, but less keel bone deformities than hens of the LB layer line [10],[11]. Dunn et al. [12] detected one significant QTL on chromosome 1 for bone index and its component traits of tibiotarsal and humeral breaking strengths in an F2 cross produced from lines of hens that had been divergently selected for bone index from a commercial pedigreed White Leghorn population.

Comparative analysis of gene expression profiles in newly developed housing systems is important to understand gene functions in chicken for adaptation and possible gene-environment interactions among layer lines. This is the first study comparing gene expression profiles of LSL and LB under the production environment of the newly developed small group housing system Eurovent German. The objectives of the present study were to characterize gene expression differences and the molecular processes associated with these differently expressed genes among the LSL and LB layer lines using the Affymetrix GeneChip® Chicken Genome Array for whole genome RNA expression profiles with 38,535 probe sets.

## Results

### Phenotype

The least-square means (LSM) of the phenotypic traits, their standard errors (SE) and their P-values for differences among the two layer lines LSL and LB are presented in Table 1. Egg quality traits were significantly influenced by the layer line, whereas LSL hens produced heavier eggs with heavier egg shells. LSL layers showed a significantly worse plumage score of the different body regions and accordingly a worse total plumage score compared to hens of the LB layer line. LB layers showed significantly higher body weights as well as higher bone weights and bone lengths than LSL. While the tibia breaking strength was approximately equal among the two layer lines, humerus bones of LB layers were significantly stronger compared to those of the LSL layer line. The mean heterophil to lymphocyte ratio (H/L-ratio) of LB layers was 2.6-fold higher than the mean H/L-ratio determined for LSL.

**Table 1:** Least-square means (LSM) with their standard errors (SE) for the phenotypic traits analysed in the two layer lines Lohmann Selected Leghorn (LSL) and Lohmann Brown (LB).

Trait	LB		LSL		LB - LSL
	LSM	SE	LSM	SE	P-value
Egg quality traits					
Egg weight (g)	62.31	0.21	63.80	0.21	<0.001
Eggshell weight (g)	6.43	0.03	6.62	0.03	<0.001
Eggshell breaking strength (N)	41.07	0.37	40.01	0.37	0.046
Albumen height (mm)	6.96	0.07	8.22	0.07	<0.001
Haugh units	81.66	0.43	89.24	0.43	<0.001
Yolk weight (g)	16.55	0.07	17.26	0.07	<0.001
Eggshell thickness (µm)	350.67	1.39	345.70	1.39	0.014
Plumage condition (1–4)					
Neck	2.23	0.07	1.43	0.07	<0.001
Back	2.80	0.11	1.14	0.11	<0.001
Wings	2.07	0.08	1.95	0.08	0.288
Tail	2.26	0.10	1.04	0.10	<0.001
Breast	1.33	0.06	1.04	0.06	<0.001
Belly	2.10	0.09	0.99	0.09	<0.001
Total	12.89	0.41	7.59	0.41	<0.001
Body weight (kg)	1.98	0.01	1.60	0.01	<0.001
Bone length (cm)					
Tibia (cm)	11.99	0.03	11.79	0.03	<0.001
Humerus (cm)	7.96	0.02	7.69	0.02	<0.001
Bone weight (g)					
Tibia (g)	11.74	0.08	9.24	0.08	<0.001
Humerus (g)	4.79	0.07	3.88	0.07	<0.001
Bone breaking strength (N)					
Tibia (N)	131.17	2.21	130.13	2.18	0.740
Humerus (N)	173.20	3.29	129.68	3.25	<0.001
H/L-ratio	0.81	0.04	0.31	0.04	<0.001

### Gene Expression Analysis

Among the two layer lines LSL and LB, 6,276 probe sets were significantly differentially expressed at a false discovery rate of P<0.05 and 6,012 of these probe sets were mapped to the chicken genome. The 6,276 probe sets could be assigned to 3,087 unique RefSeq annotated and 686 unknown, hypothetical genes. Of the 6,276 probe sets, 151 had an absolute fold change of 2-fold or greater. A total of 72 of the 151 probe sets were up- and 79 of them were down-regulated in layers of the LSL line (Table S1). These 72 highly expressed probe sets were down-regulated and the 79 down-regulated probe sets were enriched in the LB strain (Figure S1). Approximately 46% (70 transcripts) of the 151 significant probe sets were annotated in the chicken genome assembly (AmiGo, http://amigo.geneontology.org/cgi-bin/amigo/go.cgi) and 54 probe sets had a GO annotation of biological process (Table S1). A total of 18 enriched GO-terms could be identified for the probe sets with higher expression in LSL (Table 2). Immune system processes including antigen processing and presentation of peptide antigen via MHC class I with all its parents and immune response were notably observed. Most of these genes code for glycoproteins of the MHC class I complex. A transcriptional activator of the MHC class II complex, one gene encoding for the J chain of polymeric immunoglobulin molecules and one encoding for an immunoglobulin domain were also identified. Another cluster could be made of terms belonging to the membrane organization. These are cellular membrane organization, membrane invagination and endocytosis, which simultaneously is a child of vesicle-mediated transport. GO-terms endosome organization, intracellular signal transduction with small GTPase mediated signal transduction, oxidation-reduction process, regulation of cellular component organization and gene expression with its child RNA processing were also identified to be enriched. Another highly expressed gene was *SRI*, which encodes for Sorcin, a soluble resistance-related calcium-binding protein. A total of 32 enriched GO-terms were determined for the 79 down-regulated probe sets of the layer line LSL (Table 2). These included phosphorus metabolic processes with its terms phosphate metabolic process, phosphorylation and protein phosphorylation. Multicellular organismal signaling and cell-cell signaling could be assigned to the hypernym signaling. Two of the genes encode for proteins, which play a role in neurotransmitter secretion, which is a child of the equally enriched terms regulation of neurotransmitter levels, secretion by cell, signal release and neurotransmitter transport. Signal release in turn is a part of generation of a signal involved in cell-cell signaling, which is a part of the above mentioned term cell-cell signaling. Secretion by cell has two parents, namely secretion and establishment of localization in cell, which itself is a part of cellular localization. Further enriched GO-terms belonging to this section were synaptic transmission as a part of transmission of nerve impulse with its parents, neurological system process and multicellular organismal signaling, as well as regulation of biological quality and cell communication. Regulation of growth and regulation of cellular component organization both are parents of the GO-term regulation of cell growth. Another group comprised the terms regulation of anatomical structure size, its child regulation of cellular component size and finally regulation of cell size. Furthermore, following GO-terms were detected: system process, death with cell death, regulation of transcription (DNA-dependent) and regulation of RNA metabolic processes. Functions could be assigned for 94 of the 151 significant differentially expressed probe sets using online databases (Online Mendelian Inheritance in Man (OMIM), http://www.ncbi.nlm.nih.gov/omim; National Center for Biotechnology Information (NCBI), http://www.ncbi.nlm.nih.gov/) and the BLAST program of NCBI (NCBI Basic Local Alignment Search Tool, http://www.ncbi.nlm.nih.gov/blast). Thereby, a function in chicken could be found for 45 of them, whereas functions of the remaining 49 probe sets could be concluded based on the function in human. Accordingly, a total of 57 probe sets with unknown function in human and chicken respectively remained.

**Table 2:** Enriched biological processes of probe sets with different expression in comparison between LSL and LB.

GO-ID^1^	GO-Term	List Hits^2^	Total Hits^3^	P-value^4^
Upregulated in LSL				
GO:0002376	immune system process	5	470	<0.01
GO:0019882	antigen processing and presentation	4	51	<0.01
GO:0006955	immune response	4	229	<0.01
GO:0002474	antigen processing and presentation of peptide antigen via MHC class I	3	11	<0.01
GO:0048002	antigen processing and presentation of peptide antigen	3	18	<0.01
GO:0006897	endocytosis	2	85	<0.01
GO:0010324	membrane invagination	2	85	<0.01
GO:0007032	endosome organization	2	13	<0.01
GO:0016044	cellular membrane organization	2	169	0.02
GO:0061024	membrane organization	2	169	0.02
GO:0007264	small GTPase mediated signal transduction	2	263	0.04
GO:0035556	intracellular signal transduction	3	498	0.04
GO:0051128	regulation of cellular component organization	2	299	0.06
GO:0010467	gene expression	4	843	0.06
GO:0006396	RNA processing	2	323	0.06
GO:0055114	oxidation-reduction process	3	598	0.07
GO:0016192	vesicle-mediated transport	2	340	0.07
GO:0006412	translation	2	383	0.08
Downregulated in LSL				
GO:0001505	regulation of neurotransmitter levels	2	36	<0.01
GO:0003001	generation of a signal involved in cell-cell signaling	2	50	<0.01
GO:0006836	neurotransmitter transport	2	72	<0.01
GO:0007268	synaptic transmission	2	112	<0.01
GO:0007269	neurotransmitter secretion	2	20	<0.01
GO:0023061	signal release	2	50	<0.01
GO:0001558	regulation of cell growth	2	66	<0.01
GO:0008361	regulation of cell size	2	82	<0.01
GO:0032535	regulation of cellular component size	2	121	<0.01
GO:0032940	secretion by cell	2	122	0.01
GO:0019226	transmission of nerve impulse	2	133	0.01
GO:0035637	multicellular organismal signaling	2	133	0.01
GO:0090066	regulation of anatomical structure size	2	143	0.01
GO:0046903	secretion	2	158	0.02
GO:0040008	regulation of growth	2	165	0.02
GO:0007267	cell-cell signaling	2	193	0.02
GO:0008219	cell death	2	203	0.03
GO:0016265	death	2	207	0.03
GO:0003008	system process	3	451	0.04
GO:0006468	protein phosphorylation	5	970	0.04
GO:0006793	phosphorus metabolic process	6	1278	0.04
GO:0006796	phosphate metabolic process	6	1278	0.04
GO:0016310	phosphorylation	5	1031	0.05
GO:0051128	regulation of cellular component organization	2	299	0.06
GO:0023052	signaling	9	2408	0.06
GO:0065008	regulation of biological quality	4	833	0.07
GO:0050877	neurological system process	2	321	0.07
GO:0051649	establishment of localization in cell	3	598	0.07
GO:0007154	cell communication	2	361	0.08
GO:0051641	cellular localization	3	636	0.09
GO:0006355	regulation of transcription, DNA-dependent	5	1273	0.09
GO:0051252	regulation of RNA metabolic process	5	1305	0.1

1 Gene Ontology IDs of enriched terms.

2 List Hits to category: Number of differentially expressed probes on microarray belonging to specific GO-IDs.

3 Total Hits to category: Total numbers of probes on microarray belonging to specific GO-IDs.

4 P-values less the 0.1 indicate gene ontology classes that are more than 90% likely to be overrepresented.

## Discussion

The present investigation identified genes that were significant differentially expressed among the two commercial layer lines LSL and LB. In LSL hens, probe sets belonging to the GO-cluster of phosphorus metabolism were down-regulated, which may be related to an enhanced calcium requirement of LSL. In comparison to layers of the LB line, the LSL hens produced eggs with higher egg and eggshell weights. Therefore, they needed high amounts of calcium for the formation of eggshells. In order to maintain the ratio of calcium to phosphorus, layers of the LSL line may have down-regulated their phosphorus metabolism. Consequently, the intestinal phosphorus absorption has been diminished and the phosphorus concentration in blood remained constant. An increased demand of calcium may also be a reason for the higher expression of the *SRI* gene in the LSL layers. Its gene product sorcin is responsible for calcium ion binding and transport, but there are no reports about a concrete function of this protein in the chicken. The enhanced calcium requirement may have led to an increased calcium metabolism and therefore higher expression of sorcin. Brunberg et al. [6] detected an up-regulation of *SRI* in pecker LSL hens compared to victim birds and controls. The down-regulation of two transcript variants of the aspartate beta-hydroxylase (ASPH) in layers of the LSL strain may also be attributed to an enhanced calcium requirement of the LSL hens, because in eukaryotic cells this protein plays an important role in calcium homeostasis [13]. Another down-regulated gene in LSL was *ORAI1*. The ORAI calcium release-activated calcium modulator 1 is assumed to be an essential component or regulator of the calcium release-activated calcium channel complex [14].

*COMTD1* codes for a catechol-O-methyltransferase, which catalyzes the O-methylation of catechol estrogens, physiologically important catecholamines and many other catechols [15]. The best-known polymorphism in the human *COMT* gene is a functional G to A substitution, leading to an amino acid substitution of valine to methionine at codon 158 [16]. The methionine variant results in thermolability of the enzyme [17] and a 3- to 4-fold lower enzyme activity compared with the valine variant [18]. An increased risk for osteoporotic fractures and for fragility fractures has been observed in human male carriers of the Met^158^ low-activity allele with evidence for a dominant effect [16]. In the present study, expression of the *COMTD1* gene was down-regulated in LSL hens, which may have led to a decreased substrate conversion and consequently resulted in lower humerus bone breaking strengths of the LSL layers compared to layers of the LB line. The gene *ABCB1* was down-regulated in LSL and codes for a multidrug resistance protein 1 that is linked to inflammatory bowel disease, comprising Crohńs disease in humans. Patients with Crohńs disease often have low bone mineral densities and osteopenia [19],[20]. Therefore, down-regulation of *ABCB1* expression may be associated with the low humerus bone breaking strength of LSL.

The heterophil to lymphocyte (H/L) ratio is affected by stressors and can be used to measure the amount of stress imposed on layers [21]. Results of the calculation of H/L-ratio showed 2.6-fold higher ratios in LB hens compared to layers of the LSL line and H/L-ratios for LB hens indicative for long-term and high stress exposure [22]. Previous studies have shown that stress down-regulates immune responsiveness [23]–[28] and in accordance with this finding, the LB layers showed a down-regulation of genes that are related to immune system processes. However, *RCSD1* (RCSD domain containing 1), involved in stress response, was up-regulated in the LSL layers. This gene encodes for the CapZ-interacting protein (CapZIP), which is a substrate for several stress-activated protein kinases (SAPKs) [29]. Activation of SAPKs in response to a stressor may have resulted in a higher expression of its substrate CapZIP. *SGK1* codes for a serum/glucocorticoid regulated kinase 1, which plays an important role in cellular stress response. It is known that expression of *SGK1* can be acutely regulated by hormonal, mitogenic, and cellular stress signals in a cell type and stimulus-dependent manner [30]–[34]. Down-regulation of *SGK1* in LSL layers may be due to the less environmental stress imposed on them.

Nätt et al. [35] studied variations in gene expression in brains of Red Jungle Fowl, ancestor of domestic chickens and the domesticated layer line White Leghorn. Within the parentś generation they found 281 genes to be significantly (FDR-corrected P<0.05) differentially expressed, while 1674 genes could be detected for the offspring. These numbers are considerably below the results of the present study, where 6,276 probe sets were significantly differentially expressed (FDR-corrected P<0.05) between LSL and LB and 6,012 of these probe sets were mapped to the chicken genome. As could have been expected, no common genes could be found between the investigation made by Nätt et al. [35] and the current study. This indicates that gene expression is not stable among highly selected layer lines. Gene expression seems to be influenced by the selection for high egg production over many years in both layer lines, LSL and LB. Rubin et al. [36] identified selective sweeps of favourable alleles and candidate mutations that have had an important role in the domestication of the chicken and their subsequent specialisation into broiler and layer chickens. Among the 151 probe sets with differential expression between the two layer lines LSL and LB we found the gene *ANK2* to fit in one of the selective sweeps in layer lines detected by Rubin et al. [36]. This gene encodes a member of the ankyrin family of proteins, which is required for targeting and stability of Na/Ca exchanger 1 in cardiomyocytes during cardiac muscle contraction (National Center for Biotechnology Information (NCBI), http://www.ncbi.nlm.nih.gov/).

To best knowledge of the authors this is the first study that compared gene expression profiles of LSL and LB layers. In conclusion, the two layer lines significantly differed in their gene expression profiles, although they had approximately identical egg production. In particular, we identified genes encoding for proteins taking part in immune system processes and phosphorus metabolic processes. The results represent a strong basis for further improvement of immune responsiveness, egg quality and bone stability in layer lines.

## Materials and Methods

### Ethics Statement

All animal work has been conducted according to the national and international guidelines for animal welfare. Exsanguination of laying hens at the University of Veterinary Medicine Hannover was under the supervision of the Lower Saxony state veterinary office, Niedersächsisches Landesamt für Verbraucherschutz und Lebensmittelsicherheit, Oldenburg, Germany. The project has been approved by the Lower Saxony state veterinary office, Niedersächsisches Landesamt für Verbraucherschutz und Lebensmittelsicherheit, Oldenburg, as a notifiable experiment with the registration number 33.9-42502-05-11A154.

### Layer Lines and Housing System

The housing system was provided by Big Dutchman (Vechta, Germany) and was installed at the farm for education and research of the University of Veterinary Medicine Hannover (Foundation) in Ruthe. The laying period lasted from August 2009 to September 2010. Two different layer lines, Lohmann Brown (LB) and Lohmann Selected Leghorn (LSL), were equally distributed among the housing system, but not mixed within a compartment. The housing system had three tiers with five successive compartments of LSL and five of LB per tier, which were arranged in alternate order. Layers were kept in group sizes of 36 and 54 hens per compartment. According to these group sizes the compartments were 240 cm and 362 cm wide, respectively. All compartments had an equal height of 60 cm and were 135 cm deep. Hence, the available floor space per hen was 890 cm^2^. The total number of hens kept in the small group housing system was 1350. Layers were floor-reared until an age of 17 weeks. Compartments were furnished with dust baths, nest boxes with flexible curtains, claw abrasion devices, perches and a manure belt ventilator. Four perches were installed in each compartment, two of them made of white plastic and the other two of metal. They were fixed in a stepped position at different heights (9 cm and 28 cm). Additionally, the central tube for the automatic distribution of dust bathing substrate could be used for perching. The perch length for each hen was 15 cm. Figure S2 shows the general arrangement of the compartments within the housing system and the structure of a single compartment in cross section and top view. The lighting period was gradually stepped up to 14 hours per day during the first five weeks. Egg production per hen housed and average hen was approximately 77% and 84%, respectively. During the laying period, LB layers laid 304 eggs per hen housed and 314 eggs per average hen. In contrast, the number of eggs per LSL hen housed was 273 and 317 per average hen. Proportions of cracked and dirty eggs with a share of 4% each were within a tolerable range. The total mortality among laying hens was 8.4%.

### Management and Feeding

Throughout the whole laying period the hens had identical feeding and management conditions. Ad libitum feeding was distributed three to four times a day by automatic food chain. The composition of feeding stuff changed three times to give enough energy, calcium and phosphor according to the laying phase. Water was supplied ad libitum via nipple drinkers. During the rearing and laying period layers were subjected to a commonly accepted vaccination scheme for laying hens.

### Phenotypic Traits

In the 3^rd^, 9^th^ and 12^th^ laying month the egg quality traits egg weight, eggshell weight, eggshell breaking strength, albumen height, Haugh units, yolk weight and eggshell thickness were recorded for 480 eggs each.

Furthermore, the plumage condition of 480 hens each was scored in the 2^nd^, 8^th^ and 13^th^ laying month using a scale from 1 to 4. Score 1 was given for high graded damage of plumage and bare regions, score 2 meant an explicit damage of feathers and/or bare areas, score 3 completely or nearly complete feathered, but damaged feathers, and score 4 was given for a very good plumage condition with nearly no damaged feathers. Depending on the results of the assessments of the body regions head, neck, breast, belly, back, wings and tail a total plumage condition could be calculated by accumulating the scores to a total sum. In addition, the body weights of layers were measured in kilogram by placing the hens on a digital table scale.

At total of 360 out of the 480 hens that were scored for plumage condition in the 13^th^ laying month were chosen for blood sampling. Approximately 0.5 ml of blood was taken from the wing vein of each hen and within 30 minutes after sampling, one native blood smear and two methanol-fixed smears were prepared. The blood smears were stained with a modified Wright-Giemsa-staining protocol and at least 400 leucocytes, including heterophils, lymphocytes, monocytes, basophils and eosinophils were counted on one slide per hen. By dividing the relative numbers of heterophils by the relative numbers of lymphocytes the H/L-ratios were calculated.

After blood sampling the 360 hens were sacrificed by exsanguination after stunning them by rabbit punch. Within the subsequent autopsy alternately one intact left or right humerus and tibia bones were dissected and removed from muscles and tendons in order to analyse bone breaking strength. The bones were frozen at −20°C and broken within the next four weeks. Before measuring breaking strength, bones were recorded for weight and length (in g and cm, respectively). Bone breaking strength was measured in Newton (N) using a three-point-bending machine (Zwick/Z2.5/TNIS, Zwick-Roell, Ulm, Germany), which was controlled and calibrated by the technical service of Zwick-Roell in regular intervals. The punching tool exerts a constant, perpendicular force on the middle of the bone until its fracture.

### Statistical Analysis of the Phenotypic Traits

Statistical analysis was carried out using SAS, version 9.3 (Statistical Analysis System Institute, Cary, North Carolina, USA). We used the MIXED procedure to analyse egg quality traits, plumage condition, blood parameters and bone traits and calculated least-square means (LSM). Numbers of white blood cells and H/L-ratios were transformed into the natural logarithmic scale. Residuals of bone traits, logarithmized white blood cells and H/L-ratios were proved for normal distribution using the Shapiro-Wilk and Kolmogorov-Smirnow tests of the UNIVARIATE procedure of SAS. The fixed effects of group size, tier, layer line and the interactions between layer line and group size, layer line and tier were included in the statistical model for the blood parameters and bone traits. The statistical model for the egg quality traits and the plumage condition additionally comprised the fixed effects of laying month and the interaction between layer line and laying month. The individual compartments within layer line were treated as randomly distributed effects. Effects in the statistical model were tested jointly for significance using F-tests. Results of variance analysis were regarded significant when the P-values were <0.05. The residuals for all traits analysed did not significantly deviate from a normal distribution.

Statistical model for the blood parameters and bone traits:

(Log-)Y_ijklm_ = 

μ+GR_i_+TI_j_+LL_k_+LL*GR_ik_+LL*TI_jk_+comp(LL)_kl_+e_ijklm_

Y_ijklm_white blood cell numbers, H/L-ratios and bone traits

μmodel constant

GR_i_ fixed effect of group size (k = 1 to 2)

TI_j_ fixed effect of tier (l = 1 to 3)

LL_k_ fixed effect of layer line (m = 1 to 2)

LL*GR_ik_ fixed effect of interaction between layer line and group size

LL*TI_jk_ fixed effect of interaction between group size and tier

comp(LL)_kl_ randomly distributed effect of compartment within trial and layer line

e_ijklm_ random error variation

Statistical model for the egg quality traits and the plumage condition:

(Log-)Y_ijklmn_ = 

μ+GR_i_+TI_j_+LL_k_+LM_l_+LL*GR_ik_+LL*TI_jk_+LL*LM_lk_+comp(LL)_km_+e_ijklmn_

LM_l_ fixed effect of laying month

LL*LM_lk_ fixed effect of interaction between layer line and laying month

### Sampling and RNA Isolation

Based on the behaviour during handling, the plumage condition and the number of skin lesions we differentiated between stressed and unstressed layers. In consideration of the layer lines, group sizes and tiers at least two or three hens per compartment were chosen for sampling. In total samples of the cerebrum were collected from 70 layers. Samples of four different regions of the cerebrum of each hen were dissected; one from the anterior left, one from the anterior right and another two from the posterior left and right side, respectively. All samples were extracted without meninges and separately stored in reaction tubes filled with 1.5 ml RNAlater™ (Qiagen, Hilden, Germany) to fix them. Furthermore, the remained cerebrum was stuck into cryogenic tubes and was snap frozen in liquid nitrogen. The time from slaughtering to storing the samples was less then 15 minutes. After holding at +4°C for 24 hours all samples were frozen at −80°C. RNAlater™-samples of the posterior left and right side of the cerebrum were used for isolation of RNA. After thawing, approximately 50 mg tissue per sample were transferred into microcentrifuge tubes (2 ml) containing 1 ml of QIAzol® Lysis Reagent (Qiagen) and one stainless steel bead (Qiagen). Shaking them in a Tissue Lyser (Qiagen) disrupted the tissues and homogenized the lysates. Isolation of total RNA was made using the RNeasy Lipid Tissue Mini Kit (Qiagen). Steps of this method exactly conformed to the instructions of the manufacturer including the additional step of DNA-digestion. Concentration of total RNA was measured using a NanoDrop® 1000 spectralphotometer (Peqlab Biotechnologie, Erlangen, Germany).

### Synthesis of cDNA and Microarray Hybridization

Microarray analyses were performed by the Helmholtz Centre for Infection Research, Braunschweig, Germany. In total, 60 samples of the posterior left and right side of the cerebrum were used for microarray analysis. Before starting the microarray analysis quality and integrity of total RNA was assessed using the Agilent Technologies 2100 Bioanalyzer (Agilent Technologies, Waldbronn, Germany). Target preparation on Affymetrix expression arrays was made using the GeneChip® 3' IVT Express Kit (Affymetrix, Santa Clara, CA). Poly-A RNA controls were added to total RNA extractions before complimentary DNA (cDNA) synthesis according to the manufacturer protocol. A total of 500 ng RNA was reversely transcribed to initially generate first-strand cDNA as a template to create second-strand cDNA. Subsequent, in vitro transcription to synthesize antisense complimentary RNA (cRNA) and labelling with biotin-conjugated ribonucleotides (rNTPs) was performed. After purification to remove unincorporated rNTPs, salts, enzymes and inorganic phosphates 10 µg of the labelled cRNAs were fragmented and placed in a hybridization cocktail containing four biotinylated hybridization controls (BioB, BioC, BioD and Cre) as recommended by the manufacturer. Finally, samples were hybridized to an identical lot of Affymetrix GeneChip® Chicken Genome Arrays (Affymetrix) for 16 hours at +45°C. Subsequent, the arrays were washed and stained with Streptavidin Phycoerythrin using the Fluidics Station FS 400 (Affymetrix). Microarrays were scanned with the Affymetrix GCS3000 Scanner (Affymetrix) and image analysis was processed with GCOS1.2 Software Suite (Affymetrix).

### Analysis of Microarray Data

Each Affymetrix microarray contains 38,535 probe sets representing 32,773 transcripts corresponding to over 28,000 chicken genes. The array also contains 689 probe sets for detecting 684 transcripts from 17 avian viruses. For statistical analysis GeneSpring GX 11.5 Software (Agilent Technologies, Santa Clara, California, USA) was used. Normalization of the raw microarray data was performed with the Robust Multi-array Average method (RMA). After background correction the data were transformed and quantile global normalized at probe level to the median using a non-linear algorithm. Initially, raw data were filtered on expression by cut off the 20^th^ percentile to eliminate genes that are not expressed. The parameters group size, layer line and tier were analysed performing an unpaired T-test with Benjamini and Hochberg procedure as multiple testing corrections. A P-value <0.05 was considered to be significant. As a threshold to identify differentially expressed genes a 2-fold change or greater in gene expression was applied for the layer line. A gene ontology enrichment analysis was performed using the Gene Ontology (GO) Enrichment Analysis Toolkit (GOEAST, http://omicslab.genetics.ac.cn/GOEAST) with hypergeometric test and Benjamini and Hochberg as multi-test adjustment method at FDR 0.1. Additionally, the graph view of AmiGO blast server (AmiGo, http://amigo.geneontology.org/cgi-bin/amigo/go.cgi) was used to analyse relationships between the different GO-terms. Gene Ontology consists of three categories: biological process, molecular function and cellular component. Each category is structured such that specific terms are considered children of more broad terms (child-parent relationships) [37]. In the current study only categories belonging to the biological process of gene ontology are represented. We tested for gene ontology terms which were represented in inordinate or disproportionately large numbers. Significantly enriched GO-terms that contained two or more genes were used for presentation. To verify functionality of the annotated transcripts and search for physiological functions of those transcripts without a given GO-annotation we used the databases Online Mendelian Inheritance in Men (Online Mendelian Inheritance in Man (OMIM), http://www.ncbi.nlm.nih.gov/omim) and the National Center for Biotechnology Information (National Center for Biotechnology Information (NCBI), http://www.ncbi.nlm.nih.gov/). Sequence-similarity searches against the database of human were performed for transcripts which had neither GO-information nor gene symbol or accession-ID, using the BLAST program of NCBI (NCBI Basic Local Alignment Search Tool (NCBI BLAST), http://www.ncbi.nlm.nih.gov/blast).

### NCBI GEO Submission

The normalized data from the microarray gene expression experiment has been submitted to NCBI’s Gene Expression Omnibus (GEO, http://www.ncbi.nlm.nih.gov/geo/) and can be queried via GEO series accession number GSE40802 with the microarray platform accession number GPL3213.

## Supporting Information

Figure S1 **Heat map of differentially expressed probe sets among the two layer lines.** Heat map of the probe sets with absolute fold changes of 2-fold or greater detected in the comparison between the layer lines Lohmann Brown (LB) and Lohmann Selected Leghorn (LSL). The range of relative expression levels from lowest to highest is represented by the blue and red dyeing, respectively.
(DOC)

Figure S2 **Arrangement and dimensions of the compartments of the small group housing system. A** Cross section drawing of a single compartment. **B** Individual compartment for group sizes of 54 laying hens in a top view drawing. **C** Arrangement drawing of the tiers (A: first tier; B: second tier; C: third tier), layer lines (LB: Lohmann Brown; LSL: Lohmann Selected Leghorn) and group sizes (36 and 54 hens) of the small group housing system Eurovent German.
(DOC)

Table S1 **Differentially expressed probe sets between the Lohmann Selected Leghorn (LSL) and Lohmann Brown (LB) laying hens.**
(XLS)

## References

[1] Brown PO, Botstein D (1999) Exploring the new world of the genome with DNA microarrays. Nat Genet 21: 33–37. 9915498 10.1038/4462

[2] Young RA (2000) Biomedical discovery with DNA arrays. Cell 102: 9–15. 10929708 10.1016/s0092-8674(00)00005-2

[3] Yao J, Ren X, Ireland JJ, Coussens PM, Smith TPL, et al (2004) Generation of a bovine oocyte cDNA library and microarray: resources for identification of genes important for follicular development and early embryogenesis. Physiol Genomics 19: 84–92. 15375196 10.1152/physiolgenomics.00123.2004

[4] Nie H, Crooijmans RPMA, Lammers A, van Schothorst EM, Keijer J, et al (2010) Gene expression in chicken reveals correlation with structural genomic features and conserved patterns of transcription in the terrestrial vertebrates. PLoS ONE 5(8): e11990. 20700537 10.1371/journal.pone.0011990 PMC2916831

[5] Buitenhuis B, Hedegaard J, Janss L, Sørensen P (2009) Differentially expressed genes for aggressive pecking behaviour in laying hens. BMC Genomics 10: 544. 19925670 10.1186/1471-2164-10-544 PMC2785841

[6] Brunberg E, Jensen P, Isaksson A, Keeling L (2011) Feather pecking behavior in laying hens: Hypothalamic gene expression in birds performing and receiving pecks. Poult Sci 90: 1145–1152. 21597052 10.3382/ps.2010-00961

[7] Lohmann (2005) Layer Management Guide, Lohmann Brown-Classic. Lohmann Tierzucht, Cuxhaven, Germany, 2.

[8] Kjaer JB (2000) Diurnal rhythm of feather pecking behaviour and condition of integument in four strains of loose housed laying hens. Appl Anim Behav Sci 65: 331–347.

[9] Kjaer JB, Sørensen P (1997) Feather pecking behaviour in White Leghorns, a genetic study. Br Poult Sci 38: 333–341. 9347139 10.1080/00071669708417999

[10] Vits A, Weitzenbürger D, Hamann H, Distl O (2005) Production, egg quality, bone strength, claw length, and keel bone deformities of laying hens housed in furnished cages with different group sizes. Poult Sci 84: 1511–1519. 16335118 10.1093/ps/84.10.1511

[11] Weitzenbürger D, Vits A, Hamann H, Distl O (2006) Evaluation of small group housing systems and furnished cages with regard to keel bone deformations, plumage condition, claw length and body weight in Lohmann Selected Leghorn and Lohmann Brown layer lines. Arch Anim Breed 46: 89–102.

[12] Dunn IC, Fleming RH, McCormack HA, Morrice D, Burt DW, et al (2007) A QTL for osteoporosis detected in an F2 population derived from White Leghorn chicken lines divergently selected for bone index. Anim Genet 38: 45–49. 17257187 10.1111/j.1365-2052.2006.01547.x

[13] Treves S, Franzini-Armstrong C, Moccagatta L, Arnoult C, Grasso C, et al (2004) Junctate is a key element in calcium entry induced by activation of InsP3 receptors and/or calcium store depletion. J Cell Biol 166: 537–548. 15302852 10.1083/jcb.200404079 PMC1868564

[14] Feske S, Gwack Y, Prakriya M, Srikanth S, Puppel SH, et al (2006) A mutation in Orai1 causes immune deficiency by abrogating CRAC channel function. Nature 441: 179–185. 16582901 10.1038/nature04702

[15] Zhu BT, Conney AH (1998) Functional role of estrogen metabolism in target cells: review and perspectives. Carcinogenesis 19: 1–27. 9472688 10.1093/carcin/19.1.1

[16] Stolk L, van Meurs JBJ, Jhamai M, Arp PP, van Leeuwen JPT, et al (2007) The catechol-O-methyltransferase Met^158^ low-activity allele and association with nonvertebral fracture risk in elderly men. J Clin Endocrinol Metab 92: 3206–3212. 17504906 10.1210/jc.2006-2136

[17] Scanlon PD, Raymond FA, Weinshilboum RM (1997) Catechol-O-methyltransferase: thermolabile enzyme in erythrocytes of subjects homozygous for allele for low activity. Science 203: 63–65. 10.1126/science.758679 758679

[18] Chen J, Lipska BK, Halim N, Ma QD, Matsumoto M, et al (2004) Functional analysis of genetic variation in catechol-O-methyltransferase (COMT): effects on mRNA, protein, and enzyme activity in postmortem human brain. Am J Hum Genet 75: 807–821. 15457404 10.1086/425589 PMC1182110

[19] Pigot F, Roux C, Chaussade S, Hardelin D, Pelleter O, et al (1992) Low bone mineral density in patients with inflammatory bowel disease. Dig Dis Sci 37: 1396–1403. 1505291 10.1007/BF01296010

[20] Vogelsang H, Ferenci P, Woloszeznk W, Resch H, Herold C, et al (1989) Bone disease in vitamin D-deficient patients with Crohńs disease. Dig Dis Sci 34: 1094–1099. 2743850 10.1007/BF01536381

[21] Gross WB, Siegel HS (1983) Evaluation of the heterophil/lymphocyte ratio as a measure of stress in chickens. Avian Dis 27: 972–979. 6360120

[22] Gross WB, Siegel PB (1993) General principles of stress and welfare. In: Grandin T, editors. Livestock Handling and Transport. CAB International, Wallingford, U.K. 21–34.

[23] Berczi I (1998) The stress concept and neuroimmunoregulation in modern biology. Ann N Y Acad Sci 851: 3–12. 9668600 10.1111/j.1749-6632.1998.tb08969.x

[24] Dantzer R (1997) Stress and immunity: what have we learned from psychoneuroimmunology? Acta Physiol Scand Suppl 640: 43–46. 9401604

[25] Dohms JE, Metz A (1991) Stress-mechanisms of immunosuppression. Vet Immunol Immunopathol 30: 89–109. 1781159 10.1016/0165-2427(91)90011-z

[26] Glaser R, Kiecolt-Glaser JK, Malarkey WB, Sheridan JF (1998) The inﬂuence of psychological stress on the immune response to vaccines. Ann N Y Acad Sci 840: 649–655. 9629291 10.1111/j.1749-6632.1998.tb09603.x

[27] Magnusson U, Wattrang E, Tsuma V, Fossum C (1998) Effects of stress resulting from short-term restraint on in vitro functional capacity of leukocytes obtained from pigs. Am J Vet Res 59: 421–425. 9563624

[28] Sheridan JF, Dobbs C, Jung J, Chu X, Konstantinos A, et al (1998) Stress-induced neuroendocrine modulation of viral pathogenesis and immunity. Ann N Y Acad Sci 840: 803–808. 9629306 10.1111/j.1749-6632.1998.tb09618.x PMC1351103

[29] Eyers CE, McNeill H, Knebel A, Morrice N, Arthur SJC, et al (2005) The phosphorylation of CapZ-interacting protein (CapZIP) by stress-activated protein kinases triggers its dissociation from CapZ. Biochem J 389: 127–135. 15850461 10.1042/BJ20050387 PMC1184545

[30] Webster MK, Goya L, Firestone GL (1993) Immediate early transcriptional regulation and rapid mRNA turnover of a putative serine/threonine protein kinase. J Biol Chem 268: 11482–11485. 8505283

[31] Webster MK, Goya L, Ge Y, Maiyar AC, Firestone GL (1993) Characterization of sgk, a novel member of the serine/threonine protein kinase gene family which is transcriptionally induced by glucocorticoids and serum. Mol Cell Biol 13: 2031–2040. 8455596 10.1128/mcb.13.4.2031-2040.1993 PMC359524

[32] Alliston TN, Maiyar AC, Buse P, Firestone GL, Richards JS (1997) Follicle stimulating hormone-regulated expression of serum/glucocorticoid-inducible kinase in rat ovarian granulosa cells: a functional role for the Sp1 family in promoter activity. Mol Endocrinol 11: 1934–1949. 9415398 10.1210/mend.11.13.0033

[33] Alliston TN, Gonzalez-Robayna IJ, Buse P, Firestone GL, Richards JS (2000) Expression and localization of serum/glucocorticoid-induced kinase in the rat ovary: relation to follicular growth and differentiation. Endocrinology 141: 385–395. 10614661 10.1210/endo.141.1.7257

[34] Chen SY, Bhargava A, Mastroberardino L, Meijer OC, Wang J, et al (1999) Epithelial sodium channel regulated by aldosterone-induced protein sgk. Proc Natl Acad Sci USA 96: 2514–2519. 10051674 10.1073/pnas.96.5.2514 PMC26816

[35] Nätt D, Rubin C-J, Wright D, Johnsson M, Beltéky J, et al (2012) Heritable genome-wide variation of gene expression and promoter methylation between wild and domesticated chickens. BMC Genomics 13: 59. 22305654 10.1186/1471-2164-13-59 PMC3297523

[36] Rubin C-J, Zody MC, Eriksson J, Meadows JRS, Sherwood E, et al (2010) Whole-genome resequencing reveals loci under selection during chicken domestication. Nature 464: 587–591. 20220755 10.1038/nature08832

[37] Dwight SS, Harris MA, Dolinski K, Ball CA, Binkley G, et al (2002) Saccharomyces genome database (SGD) provides secondary gene annotation using the gene ontology (GO). Nucleic Acids Res 30: 69–72. 11752257 10.1093/nar/30.1.69 PMC99086

