# Supplementary material for: Differential Gene Expression from Genome-Wide Microarray Analyses Distinguishes Lohmann Selected Leghorn and Lohmann Brown Layers
Source: PLoS One. 2012 Oct 8;7(10):e46787. doi: 10.1371/journal.pone.0046787 (PMC3466173; doi:10.1371/journal.pone.0046787)
Supplement: Figure S1 — Heat map of differentially expressed probe sets among the two layer lines. Heat map of the probe sets with absolute fold changes of 2-fold or greater detected in the comparison between the layer lines Lohmann Brown (LB) and Lohmann Selected Leghorn (LSL). The range of relative expression levels from lowest to highest is represented by the blue and red dyeing, respectively. (DOC) [file pone.0046787.s001.doc]

**
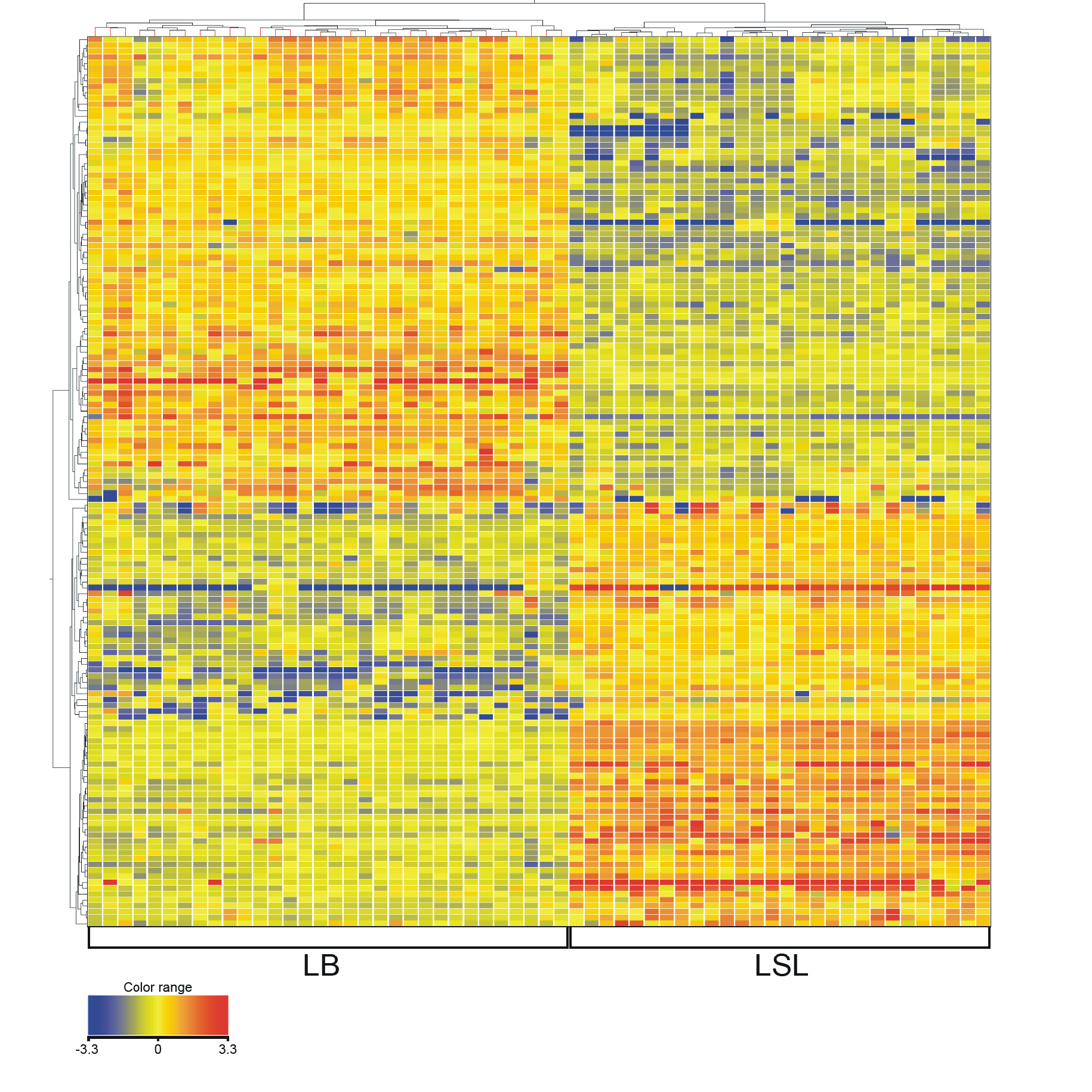
**

**Figure S1. Heat map of differentially expressed probe sets among the two layer lines.** Heat map of the probe sets with absolute fold changes of 2‑fold or greater detected in the comparison between the layer lines Lohmann Brown (LB) and Lohmann Selected Leghorn (LSL). The range of relative expression levels from lowest to highest is represented by the blue and red dyeing, respectively.
