# Supplementary material for: Differential Gene Expression from Genome-Wide Microarray Analyses Distinguishes Lohmann Selected Leghorn and Lohmann Brown Layers
Source: PLoS One. 2012 Oct 8;7(10):e46787. doi: 10.1371/journal.pone.0046787 (PMC3466173; doi:10.1371/journal.pone.0046787)
Supplement: Figure S2 — Arrangement and dimensions of the compartments of the small group housing system. A Cross section drawing of a single compartment. B Individual compartment for group sizes of 54 laying hens in a top view drawing. C Arrangement drawing of the tiers (A: first tier; B: second tier; C: third tier), layer lines (LB: Lohmann Brown; LSL: Lohmann Selected Leghorn) and group sizes (36 and 54 hens) of the small group housing system Eurovent German. (DOC) [file pone.0046787.s002.doc]

**
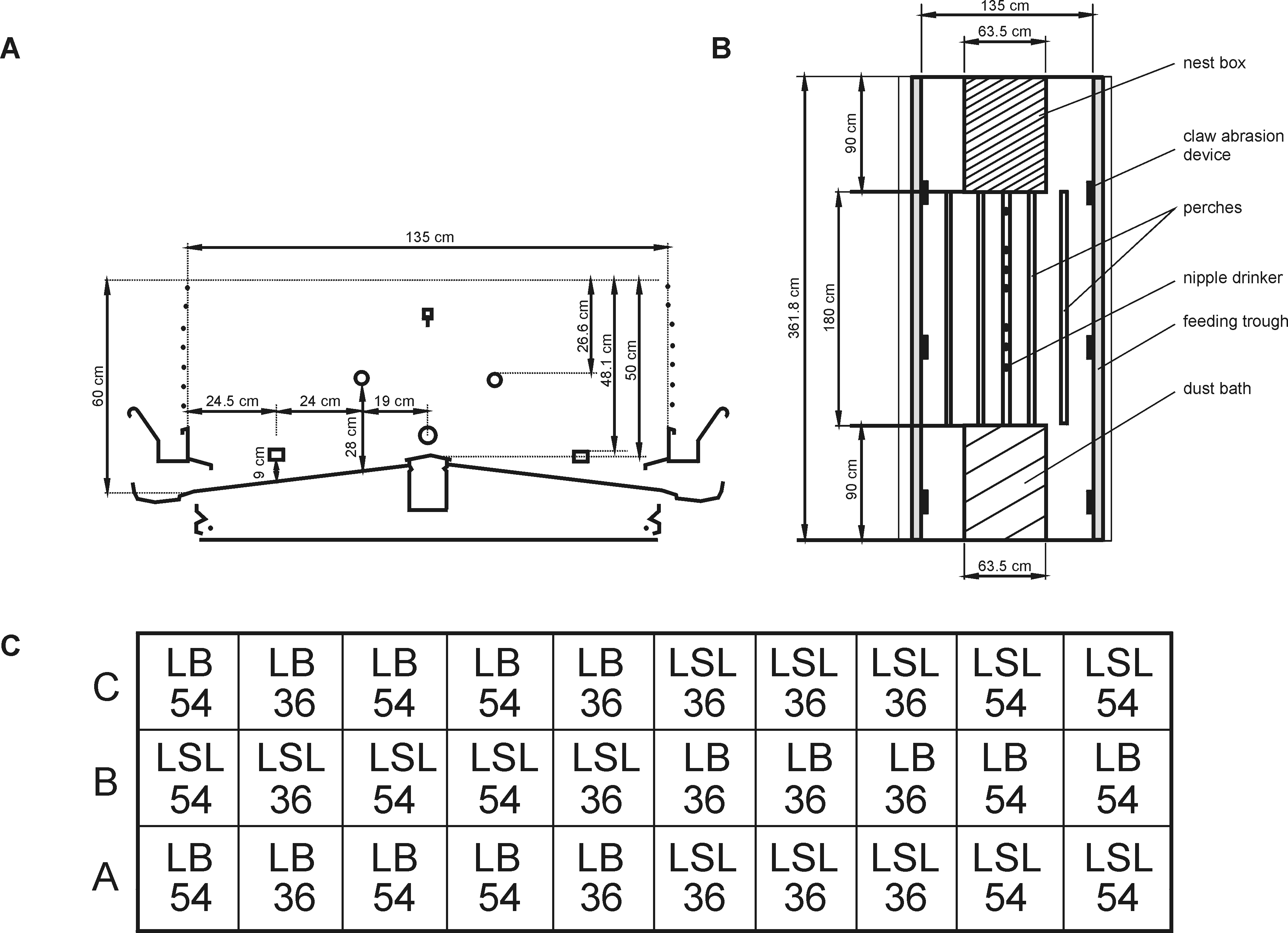
**

**Figure S2. Arrangement and dimensions of the compartments of the small group housing system. A** Cross section drawing of a single compartment. **B** Individual compartment for group sizes of 54 laying hens in a top view drawing. **C** Arrangement drawing of the tiers (A: first tier; B: second tier; C: third tier), layer lines (LB: Lohmann Brown; LSL: Lohmann Selected Leghorn) and group sizes (36 and 54 hens) of the small group housing system Eurovent German.
